# Ten simple rules for building and maintaining sustainable high-performance computing infrastructure for research in resource-limited settings

**DOI:** 10.1371/journal.pcbi.1013481

**Published:** 2025-09-22

**Authors:** Ronald Galiwango, Christopher J. Whalen, Grace Kebirungi, Mugume T. Atwine, Rodgers Kimera, Alfred Ssekagiri, Timothy W. Kimbowa, Edward Lukyamuzi, Mike Nsubuga, eLwazi ODSP, Lloyd Ssentongo, Henry Mutegeki, John M. Fonner, Frank Wuerthwein, Ari Berman, Laura B. Okalebo, Meghan McCarthy, Victor S. Kramer, Mariam Quinones, Phillip Cruz, Darrell Hurt, Maria Y. Giovanni, Nicola Mulder, Michael Tartakovsky, Jonathan Kayondo, Daudi Jjingo

**Affiliations:** 1 The African Center of Excellence in Bioinformatics and Data-Intensive Sciences, Kampala, Uganda; 2 The Infectious Diseases Institute (IDI), Makerere University, Kampala, Uganda; 3 Open Data Science Platform (ODSP) for the DS-I Africa (Data Science Initiative for Africa), University of Cape Town, Cape Town, South Africa; 4 Office of Cyber Infrastructure and Computational Biology (OCICB), National Institute of Allergy and Infectious Diseases (NIAID), National Institutes of Health (NIH), Bethesda, Maryland, United States of America; 5 Research Data and Communication Technologies Benefit Corp., Garrett Park, Maryland, United States of America; 6 Uganda Virus Research Institute (UVRI), Entebbe, Uganda; 7 Texas Advanced Computing Center (TACC), University of Texas at Austin, Austin, Texas, United States of America; 8 University of California San Diego, La Jolla, California, United States of America; 9 BioTeam, Middleton, Massachusetts, United States of America; 10 Computational Biology Division, Department of Integrative Biomedical Sciences, IDM, University of Cape Town, Cape Town, South Africa; 11 Department of Computer Science, College of Computing and Information Sciences, Makerere University, Kampala, Uganda; Dassault Systemes BIOVIA, UNITED STATES OF AMERICA

## Introduction

High-performance computing (HPC) systems are essential in health research, enabling rapid, complex data analyses for disease understanding, drug discovery, and healthcare optimization [1–3]. HPC systems comprise compute, storage and networking, facilitating communication between servers, storage devices and compute cores. They also rely on application software and skilled personnel such as systems administrators, for efficient operation.

Setting up and maintaining HPC infrastructure in Low- and Middle-Income Countries (LMICs) like Uganda is challenging due to unique challenges like limited funding, humid temperatures, and unstable power which make operations difficult. While acquiring hardware is difficult, sustaining, and managing it over time poses an even greater challenge.

**Fig 1:**
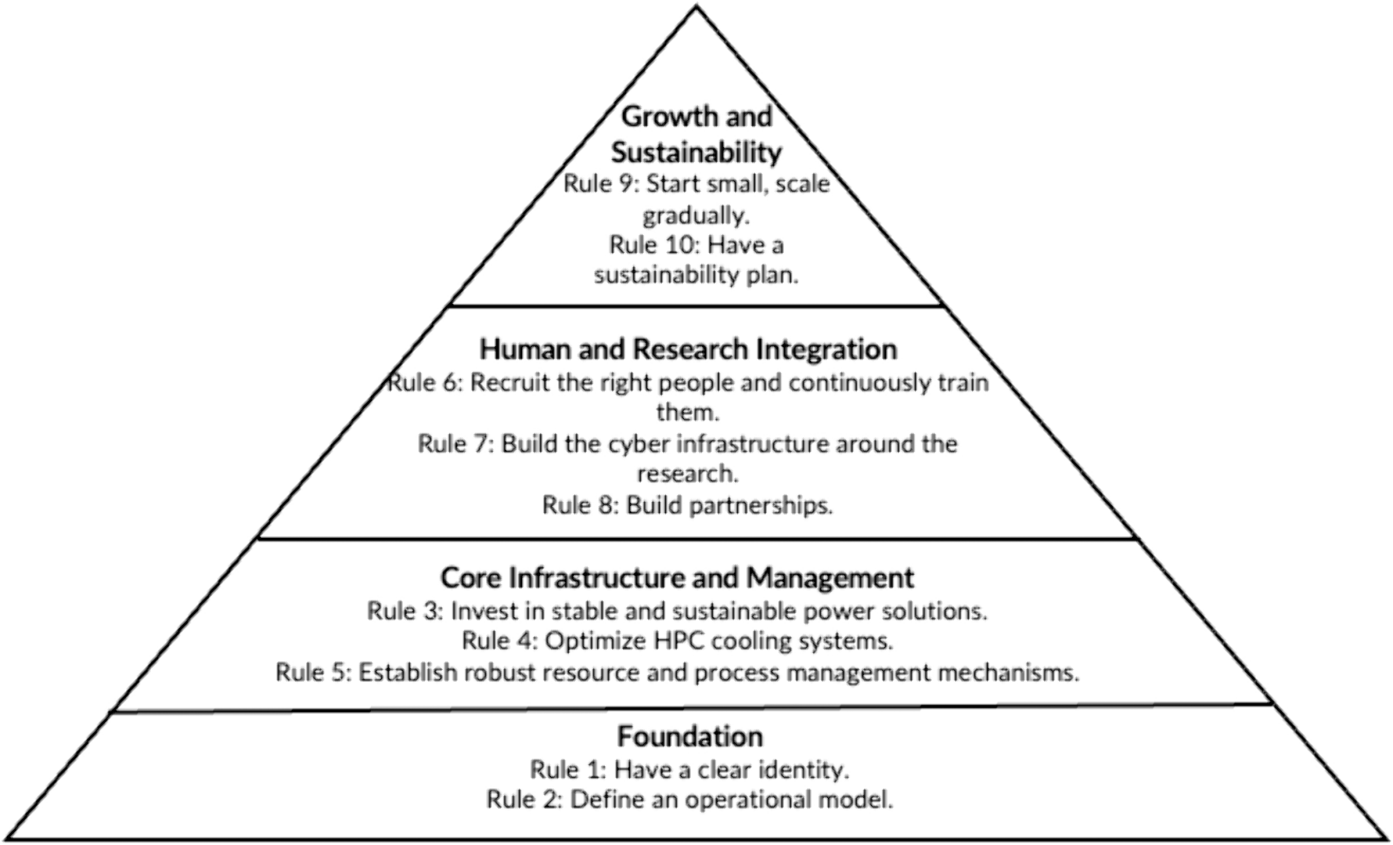
Layered pyramid summarizing the ten simple rules for sustainable HPC in resource limited settings. Abbreviation: HPC, high-performance computing.

This article shares our experiences running HPC at the African Center of Excellence in Bioinformatics and Data Intensive Sciences (ACE) Uganda [4]. When ACE-Uganda’s HPC was set up in 2019, there was no clear blueprint for managing such a system in a resource-limited setting like ours. Through trial and adaptation, we have learned valuable lessons while providing advanced computing and storage resources to researchers and bioinformatics students at Makerere University. This article distills our experiences into 10 practical rules (Fig 1) for scientists, institutions, and funders, incorporating lessons from HPC experts in well-resourced settings to adapt the system to local needs.

### Rule 1: Have a clear identity

Perhaps the most important rule is to have a clear identity in the form of a vision, a mission, strategic objectives, clearly defined metrics, and measures for success with a clearly defined roadmap, milestones, and timeline, and a charter—that describes the relationship, roles, and expectations of each partner. Make it clear what it is, who owns it, what its trajectory or strategic direction is, what you are trying to do and how to get there. This serves as a foundation for its overall success and sustainability.

*HPC is a core thematic area for ACE-Uganda, the others being Training, Research and Enhanced Visualization. Our vision for the HPC is “To be a sustainable center of excellence in large-scale computing in Africa, facilitating research and training initiatives aimed at enhancing health continent-wide.” Our strategic objectives for 2023–2028 are: 1) optimize HPC Resources by optimizing cooling and power; 2) increase the capacity of the HPC, both compute and storage; and 3) enroll new users to utilize the HPC, who in turn would provide support for operational costs and contribute to sustainability.*

### Rule 2: Define an operational model

In the context of HPC systems, an operational model is the structural framework that governs the management, maintenance, and utilization of resources to meet the computational needs of users. HPC operational models vary in structure, governance, and focus, each with distinct implications, so selecting the best fit is crucial; however, changing circumstances and funding may require adjustments over time.

The *Core Facility Model (CFM)* centralizes HPC resources within an institution, managed by dedicated IT teams and funded by user contributions, offering centralized control but limited scalability to diverse user needs. The *Research Center Model (RCM)* operates like the CFM but is tailored to a specific research domain, enhancing efficiency while limiting broader applicability and making it susceptible to shifts in research priorities and funding. The *Partnership Model (PM)* involves collaborations between different entities, e.g., government, academia, industry, etc., enabling cost-sharing and joint research initiatives but requiring complex coordination characterized by bureaucratic inefficiencies. The *Vocational Training Center Model (VTCM)* tailors HPC systems to institutional training and research needs, offering flexibility and attracting students and faculty for specialized HPC training, enhancing sustainability, but often facing resource limitations. The *Cloud HPC Provider Model (CHPM)* offers on-demand, scalable cloud computing, storage, and networking, allowing users to pay only for what they use while abstracting infrastructure management complexities, but raising security and ethical concerns, and high costs for long or computationally intensive tasks. *National or Regional Supercomputing Centers* provide large-scale infrastructure for diverse users, fostering collaboration but requiring substantial investment in hardware, energy, and management. The *Distributed Computing Model (DCM)* links geographically dispersed HPC clusters, enabling parallel or distributed computing, but demanding robust job scheduling and resource management tools. Lastly, the *Consortium Model (CM)* allows institutions to pool resources, expertise, and infrastructure to create shared HPC facilities, enhancing collaboration and cost efficiency but requiring complex governance and security management as seen in the Open Science Grid (OSG) (https://osg-htc.org/).

*ACE-Uganda operates a hybrid HPC operational model between the CFM, operating as a centralized core facility within Makerere University (an academic institution) and IDI (a research institution); an RCM focusing on health data science and bioinformatics; and a VTCM where HPC systems are tailored to the specific needs of the students, faculty, and researchers. As needed, our students also make use of the OSG facilities.*

### Rule 3: Invest in stable and sustainable power solutions

HPC research requires uninterrupted operation for extended computations, but in LMICs like Uganda, power is often expensive, unreliable, and unstable. While solar power has high upfront costs, it offers long-term savings [5]. Battery backups mitigate outages, and voltage stabilizers protect against fluctuations, ensuring system reliability.

*ACE-Uganda’s HPC is supported by a 40 KVA battery backup system which provides an extra 6 h of run time in the event of a power outage and unlikely failure of the standby generator. There is a 60 kVA Voltage Regulator—IMPR-3P60 series static electronic voltage stabilizer that provides protection against extreme temperatures and voltage fluctuations. An “online system” was recently implemented in which the HPC draws pure sine wave power from the battery inverters continuously rather than directly from the grid. This ensures that there is no fluctuation in voltage when switching between utility grid, battery, and generator power.*

### Rule 4: Optimize HPC cooling systems

HPC systems generate significant heat, necessitating efficient and sustainable cooling to protect high-value components like GPUs. Common methods include air, liquid, and immersion cooling, with high-income countries often using water-cooled chillers. Recently, model-based cooling strategies have been explored [6]. Cooling efficiency is measured using Power Usage Effectiveness (PUE), with air-cooled systems averaging 1.70+ and some immersion-cooled setups achieving as low as 1.03, reducing costs and improving system reliability [7].

The choice of cooling technology depends on infrastructure compatibility, power efficiency, and cost. Air cooling, where fans dissipate heat, is the simplest and most affordable option, but is less effective for large HPC setups. Its key advantage is the widespread availability of installation and maintenance expertise, including in LMICs. Liquid cooling [8], using water-cooled backplates, offers better heat dissipation but requires specialized infrastructure and large amounts of water [9]. Immersion cooling, the most efficient method, submerges hardware in nonconductive liquid, eliminating traditional cooling components; however demands sophisticated installation and maintenance, making it less accessible in LMICs [7].

*ACE-Uganda’s HPC is cooled by air coolers, two floor-standing Samsung Air Conditioners of 42,000 Btu/h and two wall-mounted Trane Air Conditioners of 18,000 Btu/h each, with a section of the HPC enclosed such that a small area is cooled instead of a large room that is also occupied by systems administrators.*

### Rule 5: Establish robust resource and process management mechanisms

Establish robust onboarding and account management processes to optimize HPC utilization and prevent resource wastage. Implement user-friendly application forms capturing key details like demographics, project scope, and anticipated usage. Deploy job scheduling and resource management tools to allocate resources dynamically and detect dormant or excessive usage. Set up alert monitoring systems to track system failures, alongside a ticketing system for efficient user support. Maintain continuous engagement through feedback and clear communication. Develop a flexible costing model for diverse user needs and enforce a comprehensive HPC user policy covering security, responsibilities, and operational guidelines.

*At ACE-Uganda, prospective users complete an HPC request form and must pass an onboarding quiz (≥80%) before signing the usage policy. An alert system tracks performance and notifies failures, power, or internet issues. We use SLURM for workload management and a ticketing system for user support. A fair pricing model where users contribute to operational costs, based on usage, project scope, and resource consumption, ensures financial sustainability (Rule 10) while promoting equitable access and collaboration.*

### Rule 6: The cyberinfrastructure includes people. Recruit the right people and continuously train them

A strong recruitment, staffing, and knowledge management model is essential for HPC operations. Invest in skilled personnel, including system administrators, network specialists, and user support teams. Experienced HPC consultants can secure partnerships, source equipment donations, and train staff, leveraging their expertise to avoid costly mistakes. Balancing permanent and part-time staff while leveraging HR and finance support from research arms (Rule 2 and Rule 7) optimizes resources. Establishing a structured training and retention plan ensures continuity, even if initial partners withdraw. Providing incentives and empowering local staff is crucial for long-term sustainability (Rule 10).

*At ACE-Uganda, collaborators Research Data and Communication Technologies Corporation (RDCT) manage the HPC, source partners, and develop hardware donation partnerships. NIAID provides technical expertise through systems administrators. Under-study systems administrators, recruited as interns or graduate trainees through active research grants gain mentorship from RDCT staff and senior systems administrators, and additional training through online courses and workshops. Some receive hands-on experience at the OSG School at the University of Wisconsin, supported by OSG and NIAID. This approach combines seasoned expertise with emerging talent, building a well-rounded HPC management team.*

### Rule 7: Build the cyber infrastructure around the research

Building HPC infrastructure around research establishes a sustainable, resource-efficient identity for an institution (see Rule 1), fostering innovation, attracting funding, and driving impactful discoveries. By aligning with scientific goals, HPC accelerates breakthroughs, promotes capacity building, and creates career and knowledge transfer opportunities. Supporting science as a core mission ensures long-term value, attracts diverse funding, and enables interdisciplinary collaboration, while contributing to broader sustainability goals (Rule 10) that benefit the institution and its community.

*ACE-Uganda’s HPC, developed within IDI at Makerere University College of Health Sciences, operates as a semi-autonomous entity under IDI’s Research Department, with IDI managing grants, procurement, and human resources. It generates revenue through usage by researchers within IDI, Makerere University, and the broader African research community, building on initiatives like H3Africa [* 10 *]. ACE-Uganda focuses on Antimicrobial Resistance (AMR), human genomics and cancer, and artificial intelligence (AI). Notable projects include the Uganda COVID-19 Chatbot, the HEAL project (AI-driven pandemic preparedness), the National Sickle Cell Registry [* 11 *], an Epilepsy Mobile Application, computational support for the DS-I Africa Consortium, and CAMO-NET, a global antimicrobial optimization network [* 12 *].*

### Rule 8: Build partnerships

Partnerships with tech companies (e.g., NVIDIA, AMD, Intel), academic institutions, government agencies, and the private sector provide funding, equipment donations, and users who contribute to overheads. Engaging in national and international networks fosters collaboration, while clearly defining partner roles ensures effective and sustainable cooperation (Rule 1).

*Over the last 5 years, NIAID provided the hardware applications and technical expertise for day-to-day operations. The Texas Advanced Computing Center (TACC) through NIAID donated the initial HPC set-up which was upgraded in 2024 with a more advanced and energy-efficient. Since 2022, OSG and NIAID have sponsored at least four ACE-Uganda students annually for the OSG Summer School in high-throughput computing (HTC) for data-intensive research at the University of Wisconsin, Madison. Research and Education Network Uganda (RENU) provided internet during ACE-Uganda’s establishment and continues to donate a portion of the internet currently in use, while NIAID supplies high-speed internet for ACE-Uganda and other regional sites. RENU also boosts bandwidth for key events, such as large data transfers and major training workshops. This support ensures reliable connectivity for research and training.*

### Rule 9: Start small, scale gradually

Begin with a small, efficient setup that meets immediate needs while planning for scalable expansion. Regularly monitor performance, energy use, and efficiency to refine strategies, ensuring long-term optimization and sustainability (Rule 10).

*ACE-Uganda began with a modest yet impactful setup, growing into a robust HPC center with partnerships playing a crucial role (Rule 8). TACC initially donated 40 Dell PowerEdge C8220X “Zeus” servers (640 compute cores, 1.25 TB RAM, 160 TB Synology storage, and 0.5 PB NetApp storage) though power and cooling constraints limited activation to only half of this capacity. Supported by a 30 KVA battery inverter system and RENU-donated low-bandwidth internet, usage was restricted to a few MSc and PhD bioinformatics students, with minimal software packages installed. As demand grew, additional applications and tools were deployed, all compute nodes and storage were powered, the inverter was upgraded to a 40 KVA, cooling and monitoring systems were enhanced, an alert system was set-up to monitor systems for power surges and internet outage, and NIAID supplied high-speed internet and funded key infrastructure like the 60 KVA voltage stabilizer. In 2024, TACC donated 56 Intel Xeon Phi 7250 “Knights Landing” (KNL) nodes (3,808 compute cores and 5.376 TB RAM), adding advanced, energy-efficient capacity. Plans for solar power solutions and efficient cooling underscore ACE-Uganda’s commitment to sustainability (Rule 10). This stepwise approach demonstrates how starting small and scaling strategically fostered a high-performing, resilient HPC center.*

### Rule 10: Have a sustainability plan

Sustainability is an ongoing process centered on people within the research computing ecosystem. Hardware choices impact energy efficiency, but LMICs often depend on donated systems. Regular hardware updates improve efficiency, reduce heat, and lower power consumption. A modular HPC design allows incremental upgrades in storage and compute capacity (Rule 9). Open-source software minimizes costs, while a cost-recovery model ensures users contribute to operational expenses. Diversifying funding sources supports long-term viability. A complimentary mentorship [13] and training program, such as Makerere University’s MSc and PhD in Bioinformatics hosted at ACE-Uganda, builds local expertise by training both HPC administrators and researchers to effectively utilize the infrastructure.

*Our sustainability plan includes integration into Makerere University, exploring solar power, securing research grants with budgets for supporting the HPC, and continuing to support the graduate program in Bioinformatics at Makerere University to ensure a steady pipeline of researchers. ACE-Uganda has received preliminary approval as a Makerere University Center of Excellence. Training programs like Nurturing Genomics and Bioinformatics Research Capacity in Africa (BRecA), and the Makerere University Data Science Research Training Program (MaKDaRTA) have provided scholarships to graduate students and supported trainers. As mentioned in Rule 5, we implemented a fair pricing model where users contribute to operational costs to ensure the financial sustainability while promoting equitable access and collaboration.*

## Conclusion

Technology must align with its environment, as appropriate solutions vary by context. HPC deployment in LMICs faces challenges like resource constraints and power instability. Prioritizing efficiency, collaboration, open-source tools, and capacity building can create resilient HPC systems that drive innovation. Sustainability requires ongoing adaptation, not just an initial setup. While our ten rules may seem daunting, strategic planning allows institutions to start small and scale toward a sustainable HPC system.
